# Activity and Longevity of Antibody in Paper-Based Blood Typing Diagnostics

**DOI:** 10.3389/fchem.2018.00193

**Published:** 2018-05-30

**Authors:** Clare A. Henderson, Heather McLiesh, Whui L. Then, Gil Garnier

**Affiliations:** ^1^Department of Chemical Engineering, Bioresource Processing Research Institute of Australia, Monash University, Clayton, VIC, Australia; ^2^Haemokinesis Pty Ltd., Hallam, VIC, Australia

**Keywords:** blood typing, antibody, paper diagnostic, aging, storage conditions

## Abstract

Paper-based diagnostics provide a low-cost, reliable and easy to use mode of blood typing. The shelf-life of such products, however, can be limited due to the reduced activity of reagent antibodies sorbed on the paper cellulose fibers. This study explores the effects of aging on antibody activity for periods up to 12 months on paper and in solution under different aging and drying conditions—air-dried, lyophilized, and kept as a liquid. Paper kept wet with undiluted antibody is shown to have the longest shelf-life and the clearest negatives. Antibody diluted with bovine serum albumin (BSA) protects against the lyophilization process, however, beyond 9 months aging, false positives are seen. Paper with air-dried antibodies is not suitable for use after 1 month aging. These results inform preparation and storage conditions for the development of long shelf-life blood grouping paper-based diagnostics.

## Introduction

A novel generation of paper-based diagnostics for sensing blood has recently been developed—providing a reliable, rapid and low-cost platform for blood group typing (Nery and Kubota, 2013; Then and Garnier, 2013; Then et al., 2015; Songjaroen and Laiwattanapaisal, 2016). A critical requirement for their successful commercialization is a shelf life of at least 1 year. When a diagnostic has a much longer stability than the routine product rotation period, a robust supply chain can be guaranteed.

In current prototypes (Li et al., 2012; Then et al., 2015), an antibody droplet from each of the blood typing groups of interest is delivered on the paper biosensor in some pattern, let to adsorb and preferentially dried. This results in the antibody physisorbed on the paper. Blood is then added, left to incubate, and is then washed through by saline. Positive results are indicated when agglutinated erythrocytes remain on the paper, being caught by the cellulosic network of fibers; negatives are demonstrated by the unstained paper as the unagglutinated cells wash through.

Studies have highlighted the beneficial role of paper in stabilizing and preserving antisera and blood samples (Behets et al., 1992). For a one-year shelf life of blood typing paper biosensors, the chemical and physical stability of the paper and antibody are essential. Antibody degradation can occur via several mechanisms (Wang et al., 2007) and can be deferred by additives to the antibody (Dráber et al., 1995; Su et al., 2008; Cao et al., 2017) or the paper (Huang et al., 2017). The degradation effects of high temperature, high humidity and multiple freeze-thaw cycles have been explored (Paborji et al., 1994; Wang et al., 2012). However, surprisingly little is known on the antibody-paper interaction, on the effect of the surface on the aging and adsorption morphology of antibody, or even on the degradation mechanism or shelf life of antibody solutions—and our current understanding is at best empirical (Guan et al., 2014; Wu et al., 2014; Huang et al., 2017). This in spite of a robust and reliable antibody practice and industry.

Antibodies and other biomolecular reagents are often the most expensive component of a biosensor. With low-cost diagnostics in mind, it is therefore important to use as little reagent as possible, while retaining very clear distinction between positive and negative results. Also, it is important to preserve the bioactivity of the reagents adsorbed for a long period. This is to avoid using extra antibody molecules to account for loss of activity due to aging.

This study aims at quantifying the activity and longevity of IgM blood typing antibodies physisorbed on paper in the context of blood typing diagnostic devices. Paper towel and a commercial antibody formulation were selected. There are two main objectives. The first is to quantify the effect of antibody drying mode on paper on its aging and activity behavior. Three processes on paper are compared: antibody aged after being (1) air dried, (2) lyophilized, or (3) left wet on paper. The second objective is to compare the aging and activity behavior of antibody retained on paper to those kept in solution and lyophilized /rehydrated. These objectives investigate the behavior of antibody physisorption on cellulose fibers and whether solution-based antibody storage methods can be transferred to paper diagnostics.

## Experimentals

### Materials

Epiclone Immunoglobulin M (IgM) Anti-A antibody was purchased from Commonwealth Serum Laboratories (CSL) Australia. Group A and O blood samples with ethylenediaminetetraacetic acid (EDTA) as the anticoagulant, were supplied by the Australian Red Cross Blood Service and used within 10 to 14 days post collection. Bovine serum albumin (BSA) was purchased from Sigma-Aldrich, USA, in powdered form. Analytical grade phosphate buffered saline (PBS) and NaCl were purchased from Sigma-Aldrich, USA. The PBS tablets were dissolved in MilliQ water to prepare the standard PBS buffer solution 0.9% w/v, pH 7.2–7.6. Standard Professional Kleenex paper towel (Su et al., 2012), purchased from Kimberley-Clark Australia, was used for all experiments. Eppendorf tubes, 1.5 mL, were used for storage of the solution-based antibodies during the aging process.

### Methods

Two fold serial dilutions of antibody were prepared with a 5% BSA solution in 0.9% NaCl resulting in 5 different antibody concentrations: neat (1) and 1 in 2, 4, 8, and 16 dilutions, following the standard blood testing practice (Harmening, 2012).

Paper was exposed to the antibody solutions and were left to age for periods of up to 12 months under 3 different conditions:

1. Lyophilized on paper: One centimeter squares of paper towel were soaked to saturation in the antibody solution and placed into Eppendorf tubes (ten per tube) and lyophilized.
2. As liquid on paper: One centimeter squares of paper towel were soaked to saturation in the antibody solution and stored in closed Eppendorf tubes (ten per tube). To ensure a water saturated environment, a small piece of wet sponge was placed at the bottom of each tube (the paper at no point came into contact with the sponge).
3. Air dried on paper: A 10 μL droplet of antibody solution was dispensed onto the middle of 2 cm squares of paper towel. Once completely absorbed into the paper, the sheets were air dried then stored in an air-tight plastic bag.

For comparison with aging on paper, the diluted antibody solution were also aged separately in tubes:

4. Lyophilized in tubes: Antibody solutions (200 μL) were lyophilized in Eppendorf tubes and reconstituted back to 200 μL with PBS at the time of testing.
5. As liquid in tubes: Antibody solutions (200 μL) kept in Eppendorf tubes.
6. As liquid in tubes at 4°C: As for 5, but stored refrigerated at 4°C.

Except for the refrigerated sample (method 6), all samples were stored in a temperature and humidity controlled laboratory at a constant temperature of 23°C and humidity of 50%. Lyophilizing of samples was performed on a 2.5L FreeZone® lyophilizer from Labconco Corp., Kansas City, USA. Samples were frozen in a −80°C freezer for a minimum of 30 min then immediately placed in the lyophilizer for 24 h set at −40°C with a chamber pressure of 60 mTorr.

Testing was performed periodically over the 12 months using the flow through method (Al-Tamimi et al., 2012) with three replicates per condition except for some where replicates became unusable due to various reasons:

- For antibody stored in tubes, 1 cm squares of paper towel were soaked to saturation in the antibody solution and left to air dry.
- A 3 μL drop of blood was deposited in the middle of the antibody treated paper and left to interact for 30 s.
- The test paper was then placed on blotting paper and washed three times with 100 μl of PBS drop by drop, moving the test to new blotting paper for each wash.
- The negative control tests were performed using the neat antibody.
- There were three replicates for each condition.

For a positive test, the Anti-A antibody and Group A erythrocytes bind to form agglutinates. The paper, a mixture of soft and hard wood, has cellulose fibers in the 10 to 25 μm range, giving a pore size of approximately 20 μm. Individual erythrocytes are approximately 6–8 μm in diameter and can flow through the paper during the washing step leaving an unstained piece of paper. Agglutinates, however, are larger than the pore size of the paper and get caught in the cellulose fiber network despite the washing step, leaving a dark red stain on the test paper. Measuring the intensity of the stain on the test paper indicates the amount of remaining erythrocytes and therefore the strength of the reaction.

After testing, the papers were dried overnight and then scanned on an Epson 2450 scanner. Stain intensities were measured using the software ImageJ by inverting the image and measuring the average intensity across the erythrocyte stain for each replicate, which were then averaged. A total score was calculated by summing the average scores for the full dilution titre, which was baselined to zero by subtracting the average intensity of the control negatives.

De-identified human blood samples were provided by the Australian Red Cross Blood Service, obtained with written informed consent in accordance with the recommendations of Blood Service Human Research Ethics Committee (BSHREC). This study was carried out with approval of and in accordance with the recommendations of the Monash University Human Research Ethics Committee (MUHREC).

## Results and discussion

**Figure 1:**
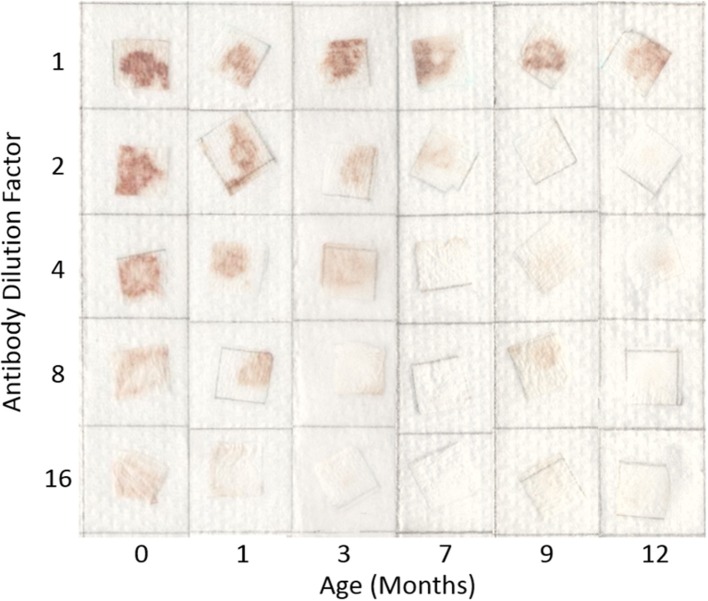
Effect of dilution and aging time on antibody bioactivity and specificity. Paper saturated with antibodies is stored wet at 23°C for up to 12 months. Antibody activity is seen to weaken with dilution and aging.

**Figure 2:**
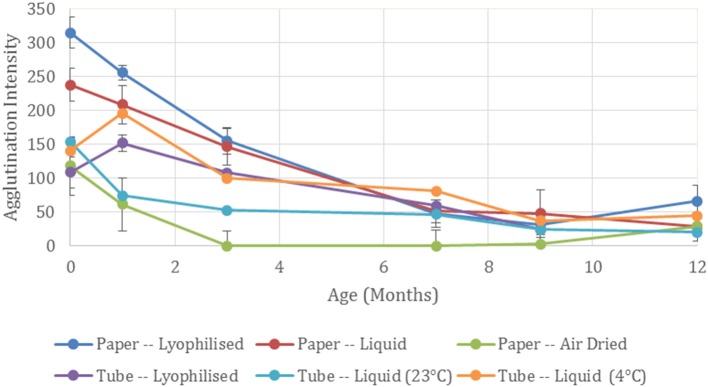
Effect of antibody storage methods on blood typing sensitivity. Summed agglutination intensity score for the full dilution titre at each time point demonstrates the antibody activity for the different storage methods aged up to 12 months.

Typical pictures of the resulting papers post-flow through are shown in Figure 1 where the reduction in erythrocyte intensity for higher dilutions of antibody is clearly seen. Each method and dilution titre was tested after being aged 1, 3, 7, 9, and 12 months. In Figure 1 it can also be seen that with age, antibody activity decreases. The effect of aging over the 12 month period for each method is shown in Figure 2, where a reduction in antibody activity is seen for all methods. The lyophilized method on paper shows the initial strongest positive results, followed closely by the method of keeping the antibody as liquid on wet paper. For the first month, these two methods give stronger results than the tests for antibody stored as a liquid. This could be due to the pieces of paper having been soaked in the antibody solution and so have more antibody. The activity of these methods decreases with age and from the 7 month mark this advantage is gone.

Air drying the antibody on paper shows the weakest initial results of the three paper-based methods. A subsequent rapid loss in activity is seen and no activity is present after 1 month of aging. This complete antibody degradation from the air-drying process may be due to a detrimental conformational change of the physisorbed antibody (Huang et al., 2017).

Although the antibody when lyophilized on paper performed the best for the paper-based methods for the first 3 months, when lyophilized in a tube, activity was less than that of the antibody stored as a liquid. The neat antibody at 7 months became difficult to resuspend, forming a gel—confirming protein denaturation (Anson and Mirsky, 1932). At 12 months the resuspension produced gelatinous globules making it unreliable for testing and no results could be recorded.

When stored as a liquid in tubes, the performance of the antibody depends on temperature with the stronger results seen when it is stored as per the manufacturer's recommendation at 4°C compared to 23°C. Activity decreased at the 7 month mark where antibody activity is comparable to the paper-based methods.

**Figure 3:**
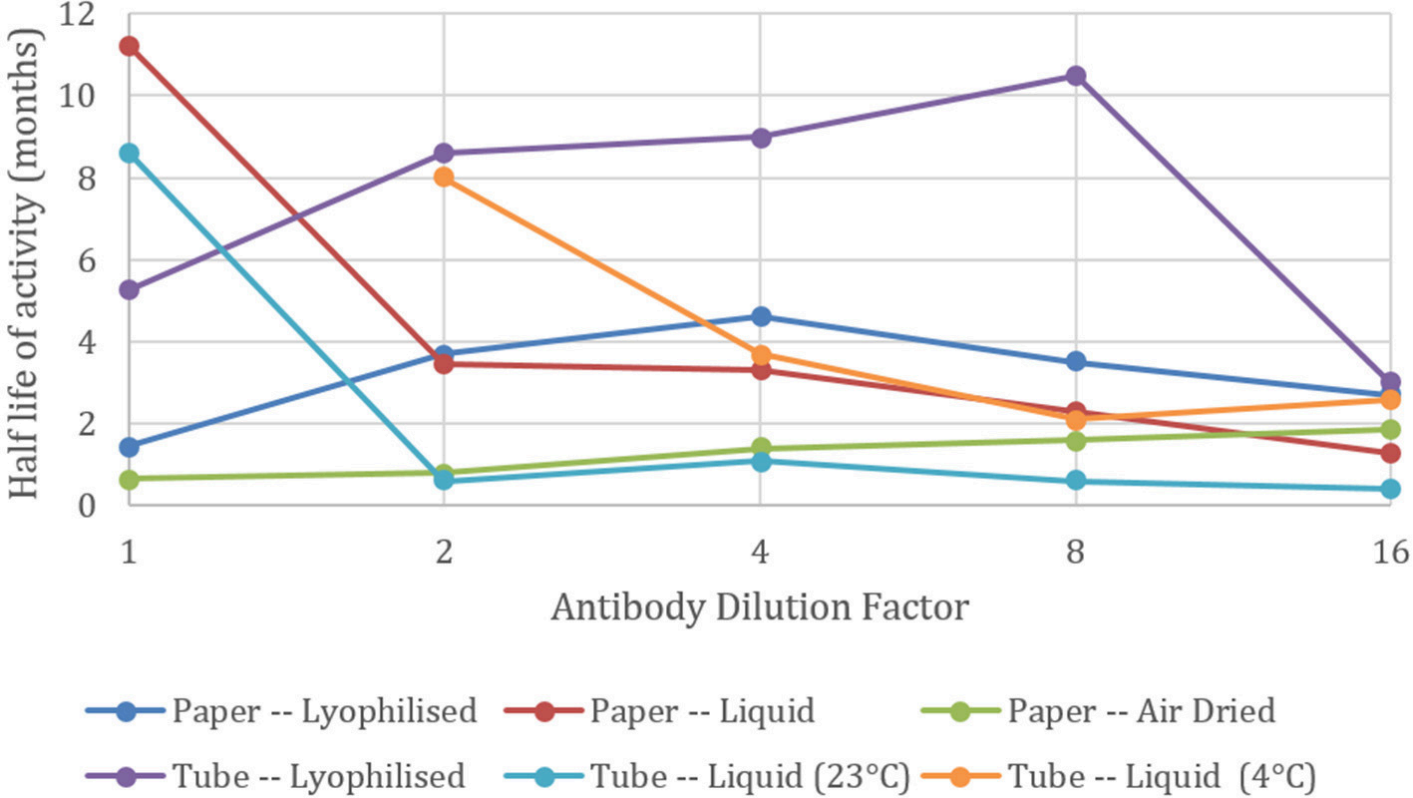
Effect of antibody dilution on blood typing sensitivity. Half-lives of agglutination intensity against neat (1) and 1 in 2, 4, 8, and 16 dilutions of antibody with a BSA-NaCl solution. Undiluted antibody solution kept as liquid in tubes at 4°C had a half-life beyond 12 months.

Not all dilutions of the antibody behaved in the same way as the general trend. Figure 3 shows the half-life for each dilution, giving the age in months for 50% loss in antibody activity. It can be seen for both the lyophilized methods, the 1 in 2, 4, and 8 dilutions perform better than the neat antibody. This suggests that the BSA from the solution is protecting the antibody from degradation in the lyophilizing process. BSA is mildly protective when air dried on paper.

**Figure 4:**
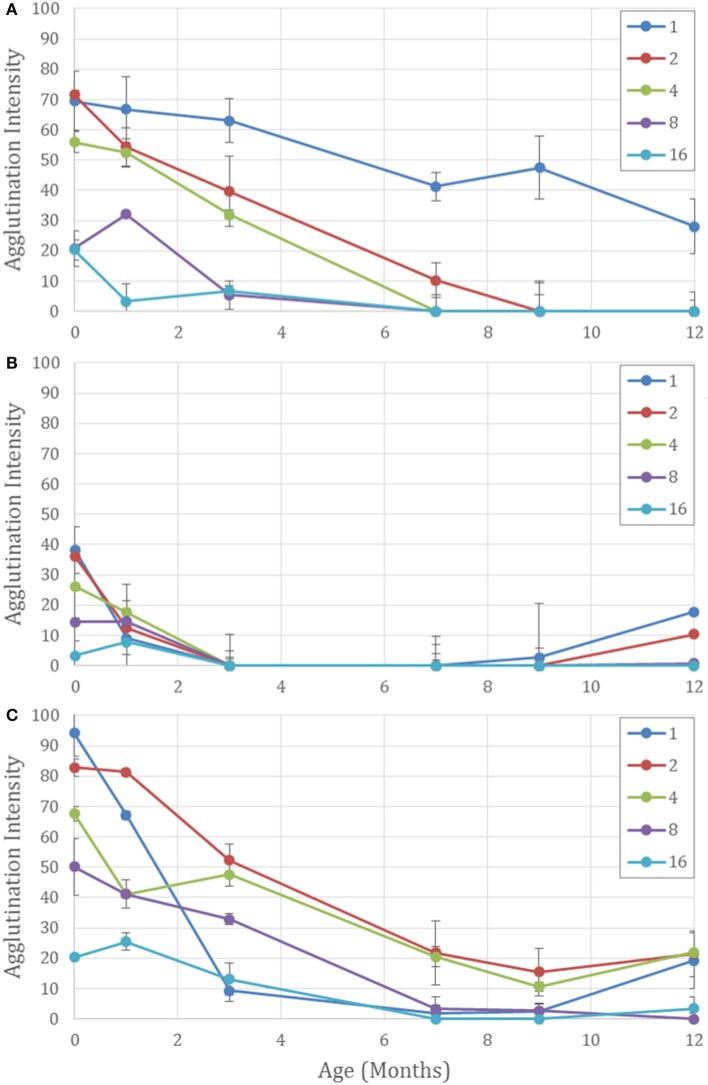
Aging effects on five different concentrations of antibody on paper aged for 12 months for Neat (1) and 1 in 2, 4, 8, and 16 dilutions with BSA-NaCl solution. **(A)** Kept wet on paper at 23°C. The undiluted antibody has the strongest activity over 12 months. Dilutions with more BSA degrade more quickly. **(B)** Air dried on paper. All antibody activity is lost at 3 months. False positives occur after 9 months—strongest for neat and lower dilutions. And **(C)** lyophilized on paper. Dilution with BSA protects the antibody against degradation and reduces false positives at 12 months for the higher dilutions.

Conversely, when the antibody is kept as a liquid in tubes or on paper, the neat antibody has the longest half-life and dilution with BSA is detrimental to the antibody's longevity (Figure 4A). All diluted samples at room temperature lost their activity after 1 month and the good performance of the general trend in Figure 2 is maintained by high activity of the neat antibody alone.

Dilutions with BSA also protected against false positives, which are apparent when the negative controls show erythrocyte staining. For the paper with air-dried antibody, false positive were seen after 9 months, and 9 months for the lyophilized on paper, but they were weaker for the higher dilutions (Figures 4B,C). It is hypothesized that the proteins in the antibody mixture prevent the red cells from adhering to the paper. After 12 months of aging denaturization of the antibody proteins may be the cause of the cells adhering to the cellulose fibers. But with higher concentrations of BSA present, protein adsorption from the BSA onto the cellulose fibers may prevent erythrocyte adhesion, allowing better flow through during the washing step and a cleaner negative result.

The paper kept wet had no false positives and gave the lowest intensity for the negative controls, leaving no visible sign of the erythrocytes retained on the paper to mask the interpretation. This allows the distinction between a weak positive and a negative result—a vital component for a diagnostic test. Moisture content and possible swelling of the cellulose fibers may prevent erythrocyte adhesion allowing the unagglutinated erythrocytes to flow through the paper, leaving a clean negative result.

The dried paper, for both the air dried and lyophilized methods, were observed to have increased hydrophobicity over time, apparent from the increased contact angle of the blood volume on the paper, impeding erythrocyte-antibody interaction. For a positive reaction to occur the antibodies must desorb from the cellulose fibers and interact with the erythrocytes to enable antigen-antibody complexation. For hydrophobic surfaces the erythrocyte suspension remained above the paper surface decreasing the surface area between the blood sample and the antibody solution. Treatment of the hydrophobized antibody paper to increase wettability could aid blood sample penetration (Tian et al., 2012). Furthermore, the washing step is hindered with liquid unable to flow through the paper, also affecting negatives. Longer interaction times were tested; however, stronger false positives resulted.

## Conclusion

The aging and activity behavior of a blood typing antibody on paper and in solution was investigated. Antibody was deposited on a paper towel and either air dried, lyophilized or kept wet; aged for periods of up to one year, and tested periodically for reaction strength. Commercially available antibody formulations and paper towel were selected for the study. Drying mode and antibody dilution are two critical variables.

The commercial antibody solutions were stable. A solution kept refrigerated at 4°C retained over 50% of its activity after 12 months when undiluted, and for 9 months at 23°C. The formulation of the antibody solution that has been optimized for antibody longevity in solution cannot be directly assumed to be the same for paper, especially when dried. The behavior of the antibody and BSA physisorption on cellulose fibers was observed.

Keeping the paper wet provided the clearest negatives and for undiluted neat antibody, had the longest half-life. Increasing paper hydrophobicity with age was observed for both the dried paper methods inhibiting the effectiveness of the test. Air dried antibodies on paper is not a suitable method for storing antibodies for a diagnostic test. Lyophilized antibody on paper provides the longest half-lives for dilutions with BSA, making the test more financially viable, however false positives were present at 12 months. This work presents references to develop paper based diagnostics meeting the shelf requirements for commercialization. It also open new horizons to stabilize antibody on surfaces.

## Author contributions

CH performed the final data analysis and wrote the manuscript including the discussion and conclusions from the experimental work and reviewed the literature. HM planned and performed all the experiments and measurements, collated the data, performed initial data analysis and contributed to the final version of the manuscript. WT assisted with experiment set-up and contributed to discussions on the project. GG supervised the project and contributed to the final version of the manuscript.

### Conflict of interest statement

The authors declare that the research was conducted in the absence of any commercial or financial relationships that could be construed as a potential conflict of interest.
